# Embedding of Genes Using Cancer Gene Expression Data: Biological Relevance and Potential Application on Biomarker Discovery

**DOI:** 10.3389/fgene.2018.00682

**Published:** 2019-01-04

**Authors:** Chi Tung Choy, Chi Hang Wong, Stephen Lam Chan

**Affiliations:** ^1^State Key Laboratory of Translational Oncology, Department of Clinical Oncology, Faculty of Medicine, The Chinese University of Hong Kong, Sha Tin, Hong Kong; ^2^State Key Laboratory of Digestive Disease, Institute of Digestive Disease, The Chinese University of Hong Kong, Sha Tin, Hong Kong

**Keywords:** gene embedding, TCGA data mining, biomarker discovery, machine learning, immunothearpy

## Abstract

Artificial neural networks (ANNs) have been utilized for classification and prediction task with remarkable accuracy. However, its implications for unsupervised data mining using molecular data is under-explored. We found that embedding can extract biologically relevant information from The Cancer Genome Atlas (TCGA) gene expression dataset by learning a vector representation through gene co-occurrence. Ground truth relationship, such as cancer types of the input sample and semantic meaning of genes, were showed to retain in the resulting entity matrices. We also demonstrated the interpretability and usage of these matrices in shortlisting candidates from a long gene list as in the case of immunotherapy response. 73 related genes are singled out while the relatedness of 55 genes with immune checkpoint proteins (PD-1, PD-L1, and CTLA-4) are supported by literature. 16 novel genes (*ACAP1, C11orf45, CD79B, CFP, CLIC2, CMPK2, CXCR2P1, CYTIP, FER, MCTO1, MMP25, RASGEF1B, SLFN12, TBC1D10C, TRAF3IP3, TTC39B*) related to immune checkpoint proteins were identified. Thus, this method is feasible to mine big volume of biological data, and embedding would be a valuable tool to discover novel knowledge from omics data. The resulting embedding matrices mined from TCGA gene expression data are interactively explorable online (http://bit.ly/tcga-embedding-cancer) and could serve as an informative reference for gene relatedness in the context of cancer and is readily applicable to biomarker discovery of any molecular targeted therapy.

## Introduction

Advances in machine learning have revolutionized our way to handle and interpret data. In particular, artificial neural networks (ANNs), a bioinspired idea to mimic the architecture of neural communication computationally, has been proven to be powerful in pattern recognition with remarkable accuracy, which allow machine to not only classify cats and dogs, oranges and apple (Krizhevsky et al., 2012; Sainath et al., 2013) but also determine good moves and bad moves in playing chess and Go (Silver et al., 2016). The same technique has also been explored in oncology to classify cancer subtypes, predict drug response and drug synergy, recognize malignant lesion in medical images so on and so forth (Menden et al., 2013; Aliper et al., 2016; Litjens et al., 2016; Araújo et al., 2017; Han et al., 2017; Khosravi et al., 2017; Mohsen et al., 2017; Preuer et al., 2017; Komura and Ishikawa, 2018; Ribli et al., 2018). However, almost all projects used supervised or semi-supervised learning method, because ANN is originally built to learn from experience and is intrinsically supervised learning, whereas unsupervised learning remains a tool mainly for exploratory data analysis and dimension reduction.

With the availability of large scale of omics data, unsupervised learning would come into sights to discover new knowledge from these existing valuable resources, i.e., to mine biological data. Conventional bioinformatic analysis, including but not limited to techniques like clustering and co-expression, has accomplished to reveal important findings from The Cancer Genome Atlas (TCGA) and International Cancer Genome Consortium (ICGC), where ANN seldom come into play. Given ANN impressing achievement in computer vision, it is anticipated to reveal information that may not be possible to find out using ordinary bioinformatic approach. To our knowledge, it has not been fully exploited whether ANN would retrieve biologically important and relevant information from these data without supervision. Some parallel unpublished works utilized variational autoencoders (VAE) system, a deep learning framework, to extract latent factors from gene expression data, and showed such latent factors are biologically relevant (Dincer et al., 2018; Way and Greene, 2018).

**FIGURE 1:**
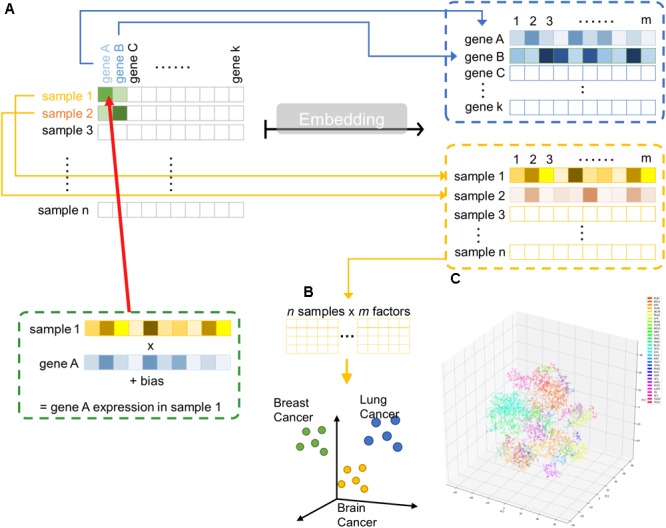
Overview of embedding system from a large gene expression dataset. **(A)** Schematic representation of entity system. Input data is a matrix of *n* samples (rows) × k genes (columns) with gene expression encoded in each cell. Through embedding, categorical variables, i.e., genes and samples, were represented as *m*-dimensional vector. **(B)** Samples are projected into *m* dimensional sample entity vector space. The embedding system learnt the feature of sample solely from input matrix, such that similar sample are clustered in close proximity. **(C)** t-SNE representation of sample entity matrix from whole TCGA RNASeqV2 dataset. 32 cancer types are labeled with different color.

Hence, we implemented a shallow two-layer ANN in the manner of word embedding (Mikolov et al., 2013a,b, 2018) to train a 50-dimension distributed representation of 20531 genes from TCGA transcriptomic dataset (Figure 1A). Word embedding is originated from natural language processing to map phrases to a continuous value vector space based on their distributional properties, which showed striking result that semantic relationship of words and phrases was preserved as distance in the vector space. We hypothesized that gene properties are also distributional, such that character of a gene can be defined by its companies in term of gene expression, and embedding can be employed to infer the relationship between genes, therefore, relevant biological information could be retrieved from embedding space (Figures 1B,C). If so, the pre-trained entity vectors could be useful to identify new biological knowledge and open up an opportunity to integrate embedding layer into other deeper neural network models.

## Materials and Methods

### Data Preparation

TCGA level3 RNASeqV2 RSEM normalized data from 36 cancer types were downloaded from The Broad Institute GDAC FireHose. COADREAD, GBMLGG, KIPAN, STES were marked as reductant with COAD and READ, GBM and LGG, KICH, KIRC, and KIRP, STAD, and ESCA, respectively. According to TCGA code tables, samples which has sample type codes starting with 1 were regarded as normal. Read counts lower than 1 were regarded as noise and replaced with 1, then the data were log2 transformed. Unless otherwise specified, data presented as cancer data was model trained from cancer only non-redundant set.

### Embedding Model

Embedding is a method to represent a categorical variable using some real numbers or mathematically defined as vector. In this case, we would like to find the numerical representation of each gene and sample by a two-layer shallow artificial neural network consisting of a single embedding layer and an output layer. The vector could be n-dimensional and the dimension of all gene and sample vector need to be equal, while we arbitrarily chose *n* = 50. To train an artificial neural network with gene expression data, the embedding matrix (composed of all gene and sample vectors) can be randomly initialized around zero. The predicted gene expression of each gene in each sample G_ai_ was defined by dot product of the respective gene vector G_a_ and sample vector S_i_ plus gene bias b_a_ and sample bias b_i_, i.e., G_ai_ = G_a_ ⋅ S_i_^T^ + b_a_ + b_i_. The neural network was trained to minimize the loss between predicted and true gene expression.

### Model Training

All weights were initialized uniformly and randomly between -0.05 and 0.05. The model was trained to minimize the mean squared loss between predicted and true gene expression using adaptive moment estimation (Adam) (Ma et al., 2018), a stochastic gradient descent method, with mini-batch size of 64. Gradient of parameters were back propagated as usual. Learning rate of the model was determined from a loss versus learning rate plot. All models were trained with three epochs. Models were all implemented in Python 3.6 using PyTorch and fast.ai library on a dedicated GPU Quadro P6000 machine hosted on Paperspace (Brooklyn, NY, United States). The training and cross validation metrics was shown in Supplementary Figure S4 and Supplementary Table S1.

### Self-Organizing Maps

Self-organizing maps (SOM) were initialized either by random or principal component analysis (PCA). Map size were set as a 1D vector with 50-component, the same dimension with gene and sample embedding vector. SOM were implemented using Python 3.6 with default setting. The quantization loss and training time was included in Supplementary Table S2.

### Visualization of Embedding Dimension

Embedding matrices were visualized by either t-distributed stochastic neighbor embedding (t-SNE) or PCA. Three dimensional t-SNE was implemented with perplexity = 5 and 50000 iterations. Three components PCA were implemented using default setting. Both t-SNE and PCA were done using scikit-learn library. The cumulative variance explained by PC1, PC2 and PC3 was included in Supplementary Table S3. Hierarchical clustering of S was performed using unweighted pair group method with arithmetic mean (UPGMA) and implemented using seaborn clustermap function.

### Gene Ontology Enrichment Analysis and Enrichment Map

Gene Ontology enrichment analysis were performed using geneSCF version 1.1-p2 (Kingma and Ba, 2014) and Gene Ontology database dated May 2018. In brief, Fisher’s exact test and Benjamini–Hochberg procedure was employed to calculate *p*-value and false discovery rate (FDR), respectively. Terms were considered statistically significant enriched if *p*-value <0.01 and FDR <0.05. Enrichment map was constructed as previously described (Subhash and Kanduri, 2016).

### Simulation of Immunotherapy Responders and Non-responders

Without access to immunotherapy responders’ gene expression data, we opt to stimulate it using TCGA dataset. To do so, we chose SKCM and LUSC to represent immunotherapy responsive cancers, while LIHC and PRAD were regarded as non-responsive cancers based on currently available knowledge of clinical response. Centroid of predicted gene expression level from responsive and unresponsive cancers were computed by multiplying the centroid of sample entity matrix with gene entity matrix. Gene with Euclidean distance smaller than threshold (0.1) with another gene was defined as close neighbors. Close neighbors of immune checkpoint proteins (*PDCD1*/PD-1, *CD274*/PD-L1, *CTLA4*/CTLA-4) present exclusively in responders were overlapped with its neighbors defined from TCGA gene entity matrix.

### Code and Data Availability

Trained embedding matrices and program codes were available and freely accessible online in https://github.com/zeochoy/tcga-embedding. The implementation of the embedding model using the code were described in the online repository. Embedding projector powered by TensorBoard can be utilized to interactively explore the entity matrices^1^. The usage of embedding projector was described in the Supplementary Figure S1. The configuration JSON files were hosted on GitHub gists.

## Results

### Preservation of Sample and Gene Relationship

To illustrate the embedding model learnt relationship between samples, cancer types could be a readily available ground truth reference. Principal component analysis (PCA) and self-organizing maps (SOM) were applied on raw log2 expression as a comparison to embedding (Table 1). Both SOM and embedding are able to capture non-linear relationship, while PCA is a linear dimension reduction method. Embedding differs from others methods by the collaborative trained sample and gene entity matrices. It is collaborative trained because the neural net is optimized by the loss between predicted and true gene expression, while the predicted gene expression is defined by sample and gene embedding matrices together (i.e., predicted gene expression is the dot product of respective sample and gene vector.). Although embedding matrices are trained collaboratively in embedding, one major drawback is its relatively longer training time (up to several hours depending on the machines and data compared to a few seconds or minutes in PCA or SOM). 50-component PCA and SOM with 50-dimensional codebook vectors were used in order to (Sainath et al., 2013) obtain component matrix with the same dimension as embedding matrices, and (Krizhevsky et al., 2012) to investigate the ability to capture underlying information from the gene expression data among different methods.

**Table 1:** Comparison between PCA, SOM, and embedding.

	PCA	SOM	Embedding
Linearity	Linear	Non-linear	Non-linear
Relation between gene and sample matrices	Not necessarily related	Not necessarily related	Trained collaboratively
Data normalization	Required	Built-in	Built-in
Training time	Short	Medium	Long

**FIGURE 2:**
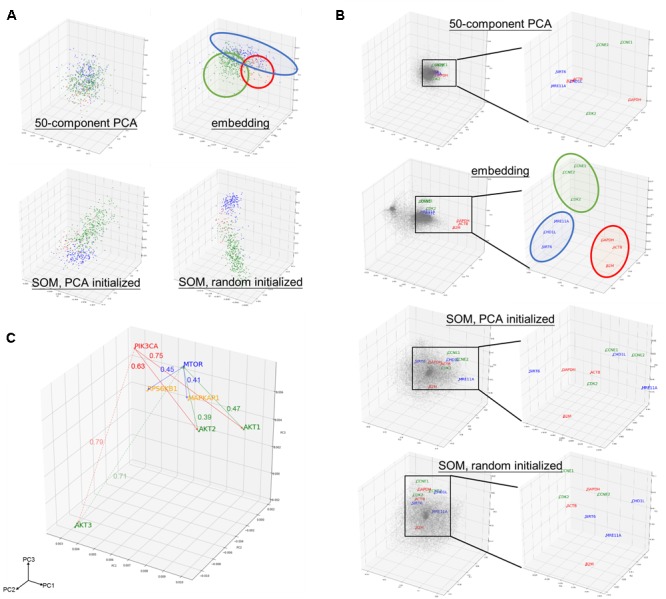
Preservation of relationship and the properties of entity model. **(A)** PCA projection of hepatobiliary and pancreatic cancers [liver hepatocellular carcinoma/LIHC (green), cholangiocarcinoma/CHOL (red), and pancreatic adenoma/PAAD (blue)] from (upper left) 50-compenet PCA projection of raw log 2 gene expression level; (upper right) sample entity matrix; (lower left) SOM with PCA initialized map, and; (lower right) SOM with random initialized map. Sample relationship are preserved by the embedding model even with dramatically reduced dimensions (20531 to 50). **(B)** PCA projection of all 20531 genes from (1st row) 50-compenet PCA projection of raw log 2 gene expression level; (2nd row) gene entity matrix; (3rd row) SOM with PCA initialized map and; (4th row) SOM with random initialized map. Housekeeping genes (*GAPDH, ACTB, B2M*) are highlighted in red. Housekeeping genes are clustered together in gene entity matrix, which showed the ability of the embedding model to understand semantic relationship between genes that not biologically and functionally related. G1 cell cycle (*CCNE1, CCNE2, CDK2*) and DNA damage response (*SIRT6, CHD1L, MRE11A*) related genes were highlighted in green and blue, respectively. PCA projection of entire gene entity matrix with PI3K/Akt/mTOR pathway components highlighted in **(C)** and a zoomed in projection with PI3K/Akt/mTOR components. PIK3CA is labeled in red; *AKT1, AKT2, AKT3* are labeled in green; *MTOR* is labeled in blue; and *MAPKAP1* and *RPS6KB1* are labeled in yellow.

Component matrix obtained from 50-component PCA (95.05% variance explained), sample entity matrix and SOM of hepatobiliary and pancreatic cancers were projected into three dimensional space and labeled with distinct color according to its cancer type as shown in Figure 2A. Samples with same cancer type were clearly clustered together after embedding and SOM, but not in 50-component PCA. It was striking to reveal that the relationships were preserved even the dimension were dramatically reduced from 20531 in log2 expression to 50 dimension embedding space. The superiority of embedding in sample relationship preservation over SOM is more obvious if all cancer types are considered as shown in Supplementary Figure S2.

The goal of embedding shall be to reflect semantic or biological relationship between genes. Housekeeping genes are suitable for such illustrative purpose, because it is not functionally interacting but semantically related. Few G1 cell cycle (*CCNE1, CCNE2*, and *CDK2*) and DNA damage response (*SIRT6, CHD1L*, and *MRE11A*) related genes were also indicated. Genes were scattered without noticeable structure before embedding, but found to harbor two distinct clusters afterward. In contrast with 50-component PCA (68.10% variance explained) of original gene expressions and SOM, related genes were clearly adjacent to each other only after embedding as shown in Figure 2B.

As aforementioned, the relationship between genes was preserved by distance in the entity space. In addition, key components of PI3K/Akt/mTOR pathway (*PIK3CA, AKT1, AKT2, AKT3, MTOR, EIF4EBP1*, and *RPS6KB1*) was taken as an example to demonstrate the vector-like property of gene entity matrix as shown in Figure 2C. *PIK3CA* had similar distance of from 0.6 to 0.7 to *AKT1* and *AKT2*, but was farther to *AKT3*. *AKT1* and *AKT2* were closer neighbors, while *AKT3* was in a different location in the entity space. Distances between *AKT1* and *AKT2* with *MTOR* were also similar, but not *AKT3*. This difference might implied a distinct expression pattern of *AKT3* and its relationship with *MTOR*. Analogous phenomenon was true for *MTOR* and its downstream effectors, *EIF4FBP1* and *RPSKB1*. One of the most compelling result of such is the ability to perform arithmetic operation on abstract concepts. For example, vector(*PIK3CA*) – vector(*AKT1*) shall be approximately equal to vector(*PIK3CA*) – vector(*AKT2*), and this property shall apply to vector(*MTOR*) – vector(*MAPKAP1*) and vector(*MTOR*) – vector(*RPS6KB1*) as well.

### Understanding the Embedding Dimensions

**FIGURE 3:**
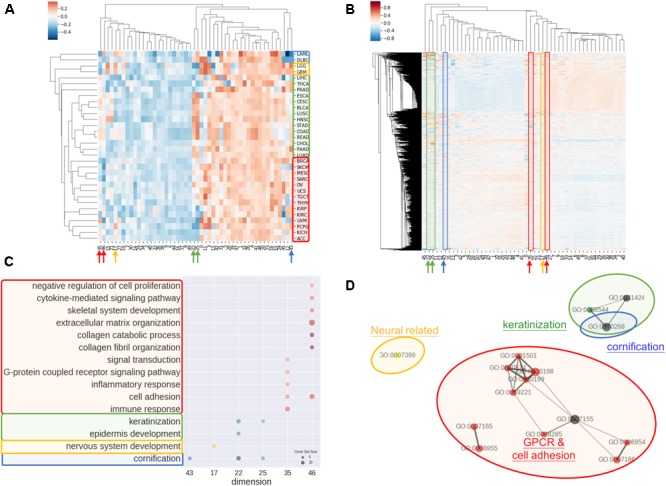
Interpretations of entity dimension. **(A)** Heatmap of cancer types (corresponding centroids) with respect to entity dimensions. Cancers were clustered into at least 4 distinct groups labeled with blue, yellow, green, and red. Group blue corresponded to blood cancers, group yellow corresponded to brain cancers, group green included gastrointestinal cancers with epithelial origin, while group red consisted of cancers with mesenchymal origin. **(B)** Heatmap of all genes with respect to entity dimensions. Gene can correspond to more than one dimension and differentially “hot” dimension between 4 sample groups are circled and indicated by arrow. **(C)** Gene Ontology (Biological Process) enrichment of top 100 genes from differentially “hot”. Notably, the “hot” dimensions were associated with distinct GO terms and related to respective group. **(D)** Enrichment map of GO term showed three apparent clusters corresponding to respective group. Node size is proportional to gene set size; edge width is proportional to overlap coefficient, while node color is consistent to group color.

Another common concern of machine learning is the difficulty to comprehend the model. In order to address the issue, we investigated the rationale of embedding dimensions. As presented in Figures 3A,B, cancer types could be differentiated from preferences in embedding dimension. Similar cancer types shared akin characteristic, thus were aggregated in a group. Four distinguishable groups highlighted in blue, yellow, green, and red were noticed.

Group blue, corresponded to blood cancers, was least weighted in dimension 12 and 43. Brain cancers were annotated as group yellow with an inclined weighing in dimension 15 and 17. Gastrointestinal cancers and a few of cancer with epithelial origins were intimately associated and labeled in green, which showed bias toward dimension 22, 25, 35, and 46. Lastly, red group consisted of endocrine cancers and the one with mesenchymal origins without obvious preferences except negatively weighted in dimension 35 and 46.

Correspondingly, biological meaning of dimensions was revealed using GO enrichment analysis as in Figures 3C,D. For example, dimension 43 was related to cornification unrelated to blood cancers. In line with group yellow, biological process concerning nervous system was over-represented in term of GO in dimension 17, while keratinization and epidermis development genes were enriched in dimension 22 and 25. Dimension 35 and 46 was linked with cell adhesion, collagen and extra-cellular matrix and G protein coupled receptors (GPCR) signaling, that is notably corresponded to be gastrointestinal cancers and less concerned in cancer with mesenchymal origins.

### Case Studies: Molecular Subtyping of Liver Hepatocellular Carcinoma Dataset

**FIGURE 4:**
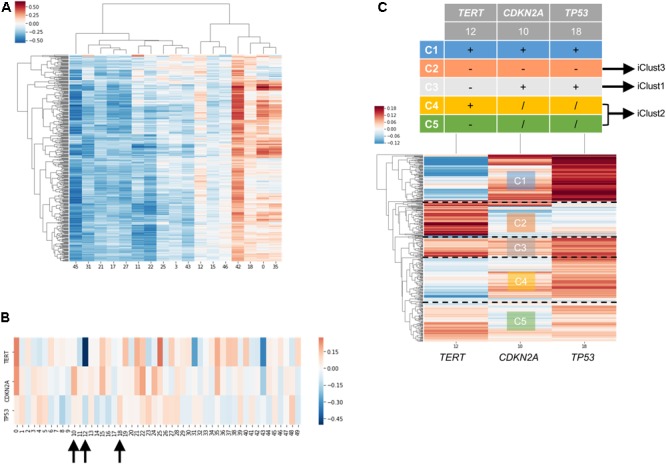
Molecular subtyping of liver hepatocellular carcinoma/LIHC using entity matrix. **(A)** Heatmap of LIHC dataset with respect to entity dimensions, only dimensions with stand deviation larger than mean stand deviation were shown. **(B)** Heatmap of three signatures genes, *TERT, CDKN2A*, and *TP53* which were previously reported to distinguish liver cancer subtypes, with respect to entity dimensions. Dimension 10, 12, 18 corresponded to *TERT, CDKN2A*, and *TP53*, respectively. **(C)** Heatmap of LIHC dataset with respect to entity dimension 10, 12, and 18 showed five distinct clusters labeled as C1-C5. C3, C4&C5, and C2 matched with iClust1, iClust2, and iClust3, respectively.

In order to examine the power of embedding, we attempted to classify molecular subtypes of liver hepatocellular carcinoma using entity matrices. Sample entity matrix revealed apparent difference between liver cancer samples in Figure 4A. Three subtypes were previously identified by TCGA Research Network on this dataset (Merico et al., 2010) using five platforms data including DNA copy number, DNA methylation, gene expression, miRNA expression and reverse phase protein lysate microarray. Integrated cluster 1, i.e., iClust1, exhibited by low *CDKN2A* silencing and low *TERT* expression, iClust2 was characterized by high *CDKN2A* silencing, while iClust3 corresponded to 17p loss. Therefore, we extracted the gene entity matrices of *TERT, CDKN2A*, and *TP53* (that is located at 17p) and selected three dimensions, 10, 12, and 18 for *CDKN2A, TERT*, and *TP53*, respectively, of disparate weights on the signature genes as illustrated in Figure 4B. The three selected dimensions were pulled out for clustering. Five groups, as annotated C1 to C5 in Figure 4C, were resolved. C3 with high *CDKN2A* and low *TERT* expression resembled iClust1, and C2 with distinguished lower weighting in dimension 18 coincided with iClust3, while substantially lower weighted in dimension 10 was similar to iClust2 characteristic. Furthermore, a cluster with heavily weighted in both dimension 10, 12, and 18 was uncovered as C1. The significance of C1 cluster is not yet known and may indicate a novel molecular subtype in liver cancer.

### Case Studies: Identification of Related Genes With Immune Checkpoint Blockade Responsiveness

**FIGURE 5:**
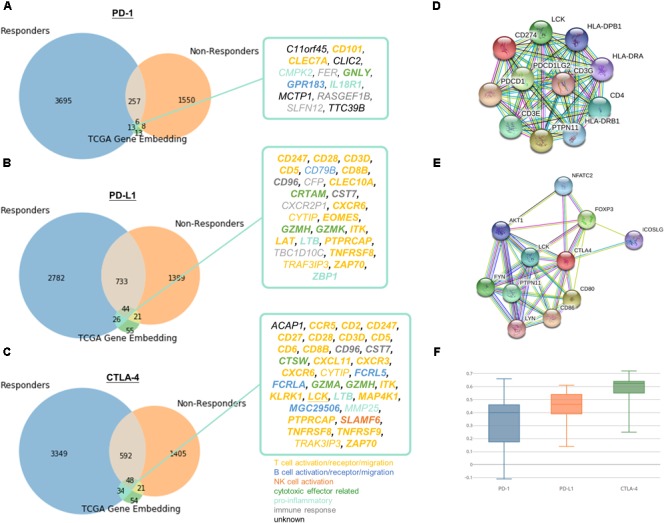
Identification of potential related genes for immune checkpoint blockade therapy responsiveness. Venn diagram of neighboring genes in simulated immunotherapy responders and non-responders with **(A)** PD-1, **(B)** PD-L1, **(C)** CTLA-4 and its corresponding neighbors in TCGA gene entity space. Functionally interacting partner (in STRING or BioGRID) are underlined. Gene relatedness supported by the literature are in bold. Genes related to T cell, B cell, NK cell, cytotoxic effector, pro-inflammatory, or immune response are highlighted in yellow, dark blue, orange, green, light blue, and gray, respectively. Known and predicted protein-protein interaction of **(D)** PD-1 and PD-L1, and **(E)** CTLA-4 were retrieved from STRING. **(F)** Boxplot of Pearson correlation between gene candidates and respective immune checkpoint protein.

To demonstrate the practical use of such embedding, responders and non-responders were simulated using TCGA data as detailed in Methods. The simulation devised as a compromise to the fact that there are no existing comprehensive transcriptomic profile of immune checkpoint therapy responders and non-responders to date. We chose two cancer types to represent immunotherapy responsive cancers (namely SKCM and LUSC) and non-responsive cancers (LIHC and PRAD), respectively. More than one cancer type is required for simulation, because we would like to single out related genes to immune checkpoint proteins instead of the differential expressed genes between cancer types. And cancer types were selected based on currently available knowledge of clinical response, such that melanoma and lung cancer are generally known to more immunotherapy responsive than liver and prostate cancer. Then, we pulled the neighboring genes with immune checkpoint proteins exclusively discovered in responder set. By overlapping the candidates with neighboring genes with PD-1, PD-L1, and CTLA-4 in learnt embedding matrices, the number of candidate genes could be successfully reduced from several thousands to fewer than twenty as shown in Figure 5. In addition, a similar simulation approach using microsatellite and *POLE* status for stratification in COAD, READ, and UCEC tumors have been included in Supplementary Data “Identification of Related Genes With Immune Checkpoint Blockade Responsiveness Stratified by Microsatellite and *POLE* Mutation Status” and Supplementary Figure S5 to illustrate the applicability of our embedding method. If comprehensive transcriptomic dataset of immunotherapy responsiveness is accessible in the future, one may pulled the differentially expressed genes to substitute the above neighboring genes method to yield more biologically accurate and relevant result. It is doubtlessly possible that not all data included in the responders set for simulation is true immunotherapy responder, therefore may hinder the usefulness and correctness of the result. However, the stimulation approach seems to be an appropriate alternative given that our limited accessibility to real life dataset, and to transfer our current clinical knowledge for potential discovery.

13 (*C11orf45, CD101, CLEC7A, CLIC2, CMPK2, FER, GNLY, GPR183, IL18R1, MCTP1, RASGEF1B, SLFN12, TTC39B*), 26 (*CD247, CD28, CD3D, CD5, CD79B, CD8B, CD96, CFP, CLEC10A, CRTAM, CST7, CXCR2P1, CXCR6, CYTIP, EOMES, GZMH, GZMK, ITK, LAT, LTB, PTPRCAP, TBC1D10C, TNFRSF8, TRAF3IP3, ZAP70, ZBP1*), and 34 (*ACAP1, CCR5, CD2, CD247, CD27, CD28, CD3D, CD5, CD6, CD8B, CD96, CST7, CTSW, CXCL11, CXCR3, CXCR6, CYTIP, FCRL5, FCRLA, GZMA, GZMH, ITK, KLRK1, LCK, LTB, MAP4K1, MGC29506, MMP25, PTPRCAP, SLAMF6, TNFRSF8, TNFRSF9, TRAF3IP3, ZAP70*) genes were speculated to be related to PD-1, PD-L1, and CTLA-4, respectively. Pearson correlation coefficients ranged from -0.11 to 0.72 between candidates and respective immune checkpoint protein in responders dataset (Figure 5F). The relatively large range of correlation coefficients reflected the embedding model do not only discover co-expressed pairs, but also other related genes in biological or semantic sense. Only 1 of the candidates, *LCK*, was functionally interacting partner in STRING or BioGRID.

Of note, the relations of 75% of the pairs were supported by the literature (Supplementary Tables S4–S6). 11 out of 16 remaining 25% were mainly contributed to immune response but lacking direct evidence to support their association between the checkpoint proteins and candidates (*CD79B, CFP, CMPK2, CXCR2P1, CYTIP, FER, MMP25, RASGEF1B, SLFN12, TBC1D10C, TRAF3IP3*), while 5 genes (*ACAP1, C11orf45, CLIC2, MCTP1, TTC39B*) were poorly characterized with unknown or limited information on their biological functions. We speculated that 16 novel genes might be effector genes corresponding to antitumor immunity and act as indicators for successful checkpoint blockade therapy. On the other hand, most of the potential genes (*CCR5, CD101, CD2, CD247, CD28, CD3D, CD5, CD6, CD8B, CLEC7A, CLEC10A, CXCL11, CXCR3, CXCR6, CYTIP, EOMES, ITK, KLRK1, LAT, LCK, MAP4K1, PTPRCAP, TNFRSF8, TNFRSF9, TRAK3IP3, ZAP70*) were involved in T cell signaling (including T cell activation, T cell receptor signaling and T cell migration), others were engaged in B cell (*CD79B, FCRL5, FCRLA, MGC29506, GPR183*), NK cell signaling (*SLAMF6*), cytotoxicity (*CRTAM, CTSW, GNLY, GZMA, GZMH, GZMK*) or known to be pro-inflammatory (*CMPK2, IL18R1, LTB, MMP25, ZBP1*). Remaining genes were either recognized to be involved in immune response but with undetermined biological function (*CD96, CFP, CST7, CXCR2P1, FER, RASGEF1B, SLFN12, TBC1D10C*) or with unknown role in immune system at all (*ACAP1, C11orf45, CLIC2, MCTP1, TTC39B*).

## Discussion

We applied embedding, an unsupervised machine learning method originally used for natural language processing, to mine expression data. By embedding, sample and gene relationship are resolved as evidenced by the model ability to preserve known entities, such as cancer types and semantic meaning of genes. The underlying mechanism of the model is easily understandable instead of depicted as black-box in other machine learning or ANNs (Cancer Genome Atlas Research Network. Electronic address: wheeler@bcm.eduA and Cancer Genome Atlas Research Network, 2017), while a straightforward posterior over-representation analysis or enrichment analysis is enough to determine the biological meaning of embedding dimensions. On top of that, the model could be exploited to spot previously undiscovered function or related pathway of a gene by inspecting its coordinates in embedding space. It is possible because each gene is not assigned to a particular system initially, but to embedding space that corresponds to many systems. One may imagine the input dataset as a collection of experimental results, in which certain genes were disrupted in each sample, in particular, the case of cancers, as traditional knockdown/overexpression assay and its resulting gene expression change was recorded by RNA sequencing. In the sense, it is easier to interpret the process undertaken by embedding and imagine the power of such model.

A major advance made by embedding is its capability to learn without the need of existing knowledge base. Similar works had been done by inferring ontologies from similarities matrix of molecular networks either as unsupervised or semi-supervised (Dutkowski et al., 2013; Kramer et al., 2014; LeCun et al., 2015; Li and Yip, 2016; Paul and Shill, 2018). However, both studies worked on similarities matrix rather than raw expression data. Even further improvements have been made on threshold setting and tree construction, it still describes linear relationship only. On the contrary, the relationships learnt using embedding are non-linear, because its findings cannot be explained solely using correlation coefficient. This non-linearity potentially enables embedding to surpass conventional bioinformatic analysis approach in discovering biological data relationships, such as biclustering and co-expression provided by Oncomine, cBioPortal, and TCGAbiolinks. Furthermore, a recent landmark paper (Sykacek, 2012) on using a visible neural network to model yeast cell system has implicated the potential advantage of such computational inferred data (i.e., CliXO (Dutkowski et al., 2013) or entity matrix) over manually curated database [GO (The Gene Ontology Consortium, 2017)] by experts in discovering new biological process.

Although data preparation is minimal for embedding comparing with models working on statistic (similarity matrix), data shall be critically chosen because gene entity matrix is intrinsically sensitive to the input data as demonstrated in Supplementary Data “Robustness of Embedding Model Towards Different Sample Types” and Supplementary Figure S3. For example, if you want the model to learn the gene network of hepatobiliary pancreatic cancers, you shall only input HBP cancers without normal control samples. Otherwise, the model could learnt the connections from normal data as well and overwhelm the desired result.

Still, the implementation of embedding is simple but its result is valuable. It is different from other machine learning or deep learning model, because prediction is not our interest. Compared with existing method to map latent space of expression data using VAEs (Dincer et al., 2018; Way and Greene, 2018), embedding is concise in architecture and easier to train but also achieve biologically relevant entity space. Parallel work using the same technique but trained on datasets from GEO database also demonstrated the trained matrix reflected functional relationship (Du et al., 2018). However, we suggested the representation may go beyond functional interactions to capture semantic understanding of genes, such as housekeeping genes. In addition, the use of embedding could be plentiful. We demonstrated the gene entity matrix could serve as an immediate reference to gene relationships to prioritize or single out gene lists, and the sample entity matrix could be further exploited for molecular subtyping. It is compelling to apply the same method to identify biomarkers of diseases or synergistic targets of a drug treatment. One may utilize the predictive power of the model to extrapolate unknown or missing gene expressions values using a subset of gene expression profile, such as targeted RNA sequencing. It might seem irrelevant or impossible at first sight, but the machinery behind gene embedding is a technique called collaborative filtering. Collaborative filtering is a widely adopted recommender system to make predictions of users’ interest by their preferences as seen in Google, Facebook, Twitter and Netflix (Das et al., 2009; Su and Khoshgoftaar, 2009; Gupta et al., 2013) etc. Apart from these, embedding could be coupled with other neural network architecture that trains together with the neural network or incorporate the pre-trained entity matrices into the new model.

## Author Contributions

CC contributed to the implementation, analyzed the data, and wrote the manuscript. CW and SC conceived the project. All authors contributed to critical review of the manuscript.

## Conflict of Interest Statement

The authors declare that the research was conducted in the absence of any commercial or financial relationships that could be construed as a potential conflict of interest.

## Footnotes

1 http://bit.ly/tcga-embedding-cancer
