# Supplementary material for: Embedding of Genes Using Cancer Gene Expression Data: Biological Relevance and Potential Application on Biomarker Discovery
Source: Front Genet. 2019 Jan 4;9:682. doi: 10.3389/fgene.2018.00682 (PMC6329279; doi:10.3389/fgene.2018.00682)
Supplement: Supplementary file 1 [file Data_Sheet_1.docx]

Supplementary Material

Embedding of genes using cancer gene expression data: biological relevance and potential application on biomarker discovery

Chi Tung Choy ^1^, Chi Hang Wong* ^1^, Stephen Lam Chan* ^1,2^

^1^ State Key Laboratory of Translational Oncology, Department of Clinical Oncology, Faculty of Medicine, The Chinese University of Hong Kong, Sha Tin, Hong Kong
^2^ State Key Laboratory of Digestive Disease, Institute of Digestive Disease, The Chinese University of Hong Kong, Sha Tin, Hong Kong

*** Correspondence:**Dr Chi Hang Wong
[eric@clo.cuhk.edu.hk](mailto:eric@clo.cuhk.edu.hk)
Dr Stephen Lam Chan
[l_chan@clo.cuhk.edu.hk](mailto:l_chan@clo.cuhk.edu.hk)

# Supplementary Data

## Robustness of embedding model towards different sample types

One major question is whether the embedding model is robust or not, such that how the learnt entity matrix changes if the data is mixed. We examined the robustness by comparing parameters correspond to individual gene and sample as a whole, owing to the fact that direct comparison of embedding dimension would be inappropriate and fairly impossible. The reason is that different training models were initiated randomly, therefore dimension 0 in model A would not be equal to or correspond to dimension 0 in model B. To our best, we examined the gene entity matrix mean and standard deviation distributions between cancer and normal samples. Gene entity matrices were sensitive to mixed sample types. In other words, gene embedding would change if we trained our model using different sample types.

**1.2 Identification of related genes with immune checkpoint blockade responsiveness stratified by microsatellite and *POLE* mutation status**

To further support the applicability of our embedding method, we extended the simulation approach to categorize possible responders and non-responders of immunotherapy by microsatellite and *POLE* mutation status. The microsatellite and *POLE* mutation status of tumors in COAD, READ and UCEC dataset were retrieved from National Cancer Institute Genomic Data Commons Data Portal through TCGAbiolinks package (version 2.10.0) and cBioPortal respectively. Tumors labeled as MSI-H (high level of microsatellite instable) or carrying mutated *POLE* were considered as responders group, while tumors labeled as MSS (microsatellite stable), MSI-L (low level of microsatellite instable), MSI-I (intermediate level of microsatellite instable) or carrying wild type *POLE* were considered as non-responders group. The exact workflow as described in section 2.7 was applied on the data, where centroid of predicted gene expression level from responder and non-responder group were computed, close neighbors of immune checkpoint proteins (*PDCD1*/PD-1, *CD274*/PD-L1, *CTLA4*/CTLA-4) present exclusively in responders were overlapped with its neighbors defined from TCGA gene entity matrix. The TCGA embedding matrix could also reduce the number of candidate genes from a thousand to fewer than ten genes as shown in Supplementary Figure 5. Most of (14 out of 17 candidates) the candidate genes identified were related to immune process, including T cells signaling (*CD5, CD6, CXCR3, CYTIP, FOXP3, GFI1, LAT, PYHIN1, UBASH3A, ZAP70*), B cells signaling (*MCOLN2*), pro-inflammatory *(PLA2G2D*) or others immune response (*APOBEC3D* and *GPR114*).

# Supplementary Figures and Tables

##
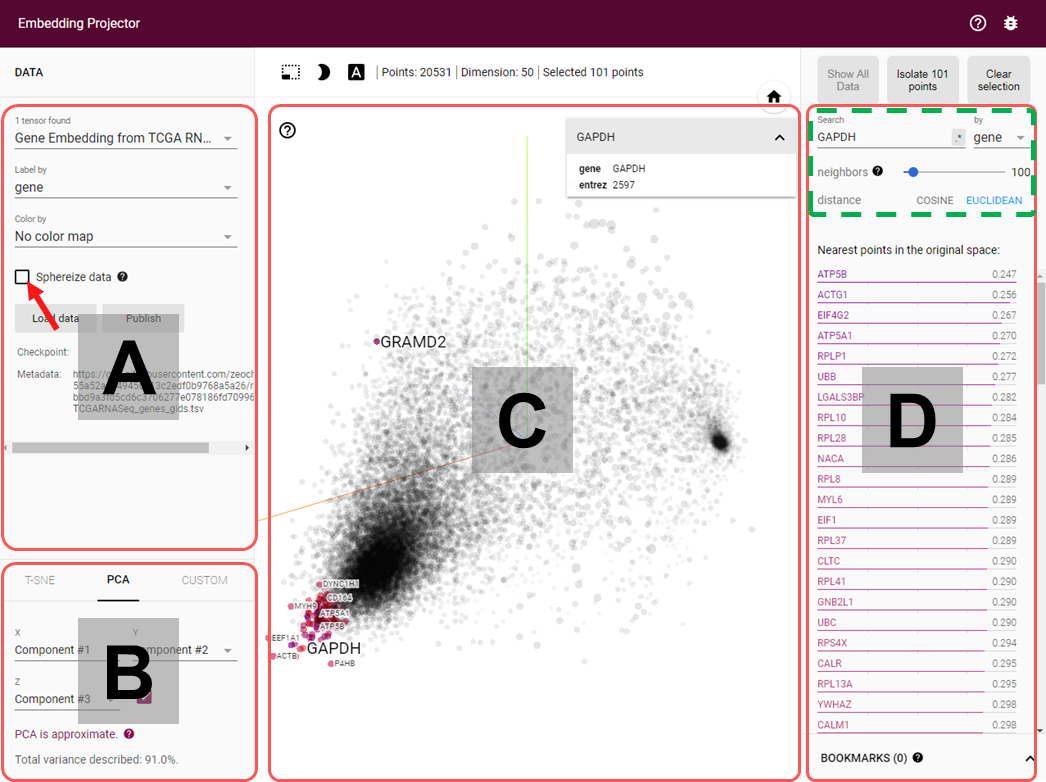
Supplementary Figures

**Supplementary Figure 1**. Screenshot of interactive web-based embedding projector. User can easily pull out related genes in term of TCGA embedding matrices using the embedding projector and explore the neighbors of target genes. Briefly, embedding projector is a tool developed by TensorBoard to interactively visualize embeddings. The projector consists of four panels. (A) is the data panel that document the user input data (called tensor) and choose how the data points is colored and labeled. There is an option to sphereize data (standard normalization) as indicated by red arrow. (B) is the projections panel where one may choose to visualize the data by t-SNE or PCA. (C) is the viewport where zooming and positioning can be controlled intuitively. User can also hover over or click on the data point to show its gene name and entrez ID. (D) is the inspector panel where user can search for a particular gene and a list of nearest neighbors. Green box indicates the controller inside the inspector panel. User can control the number of nearest neighbors to show and which distance metrics (cosine or euclidean) to use for finding nearest neighbors.

**
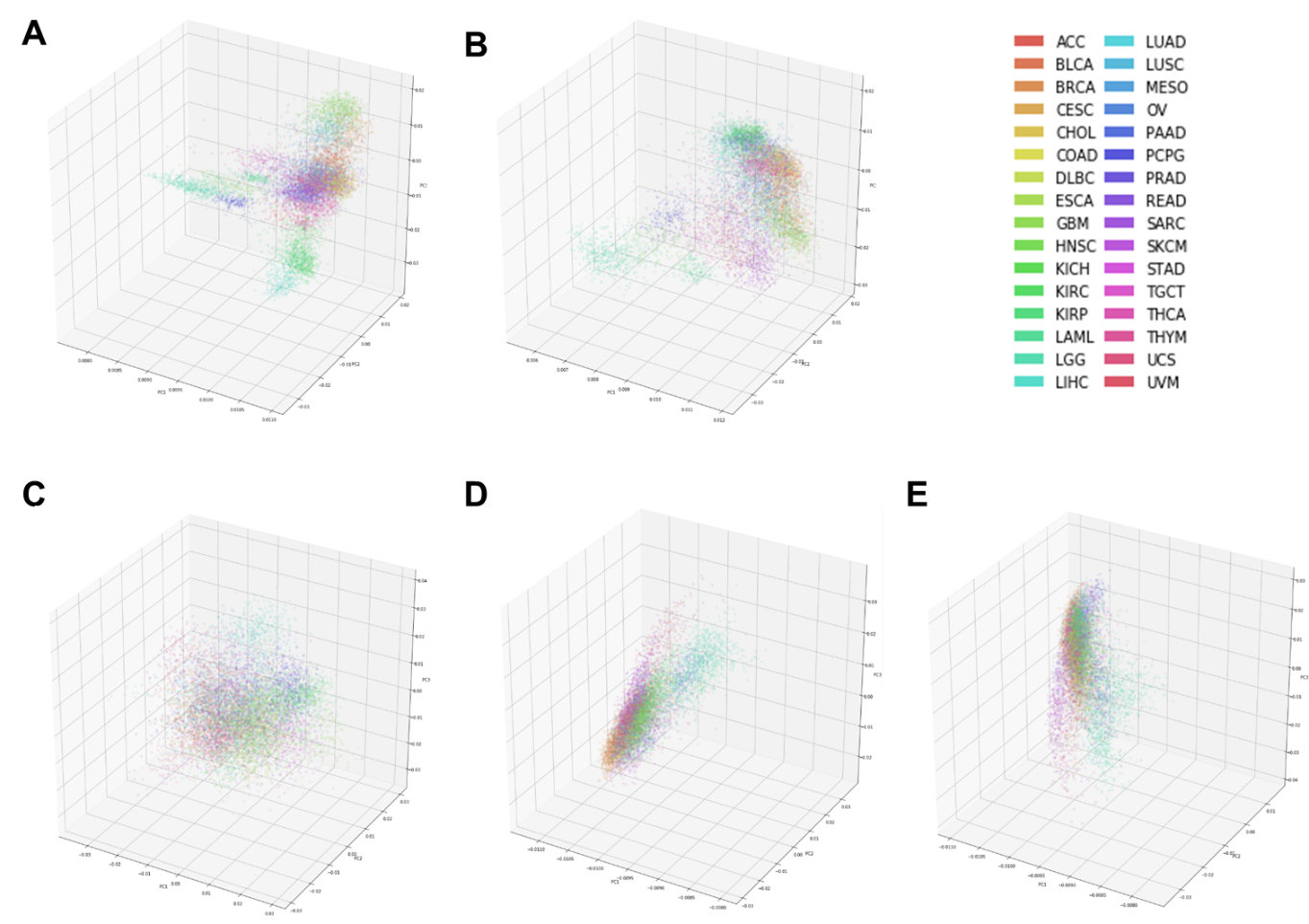
Supplementary Figure 2.** PCA projection of (A) raw log2 gene expression, (B) sample embedding matrices, (C) 50-compenent PCA projection of log2 gene expression, (D) SOM initialized by PCA and (E) SOM initialized randomly.

**
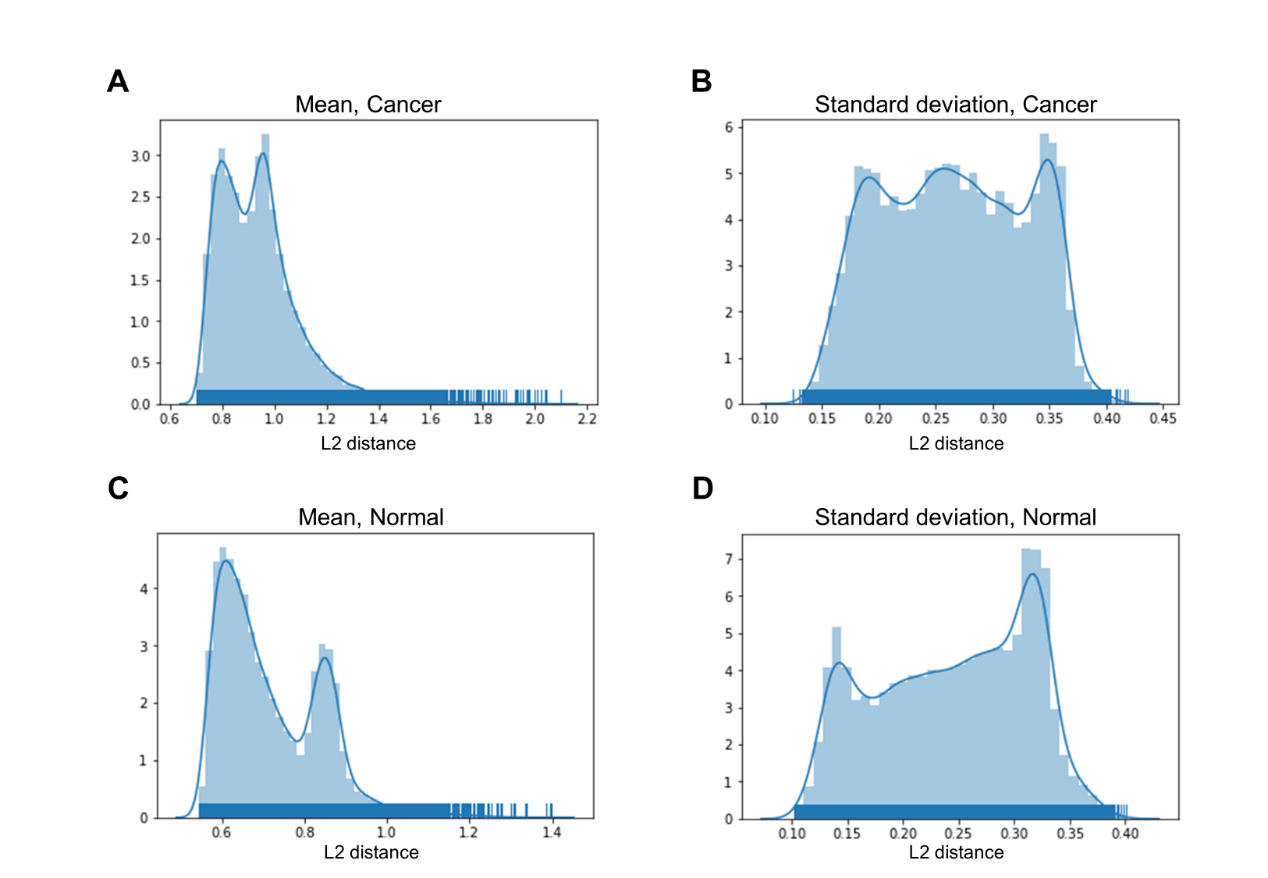
**

**Supplementary Figure 3.** Euclidean distance (A) mean and (B) standard deviation distribution of cancer set; and Euclidean distance (C) mean and (D) standard deviation distribution using normal samples data.


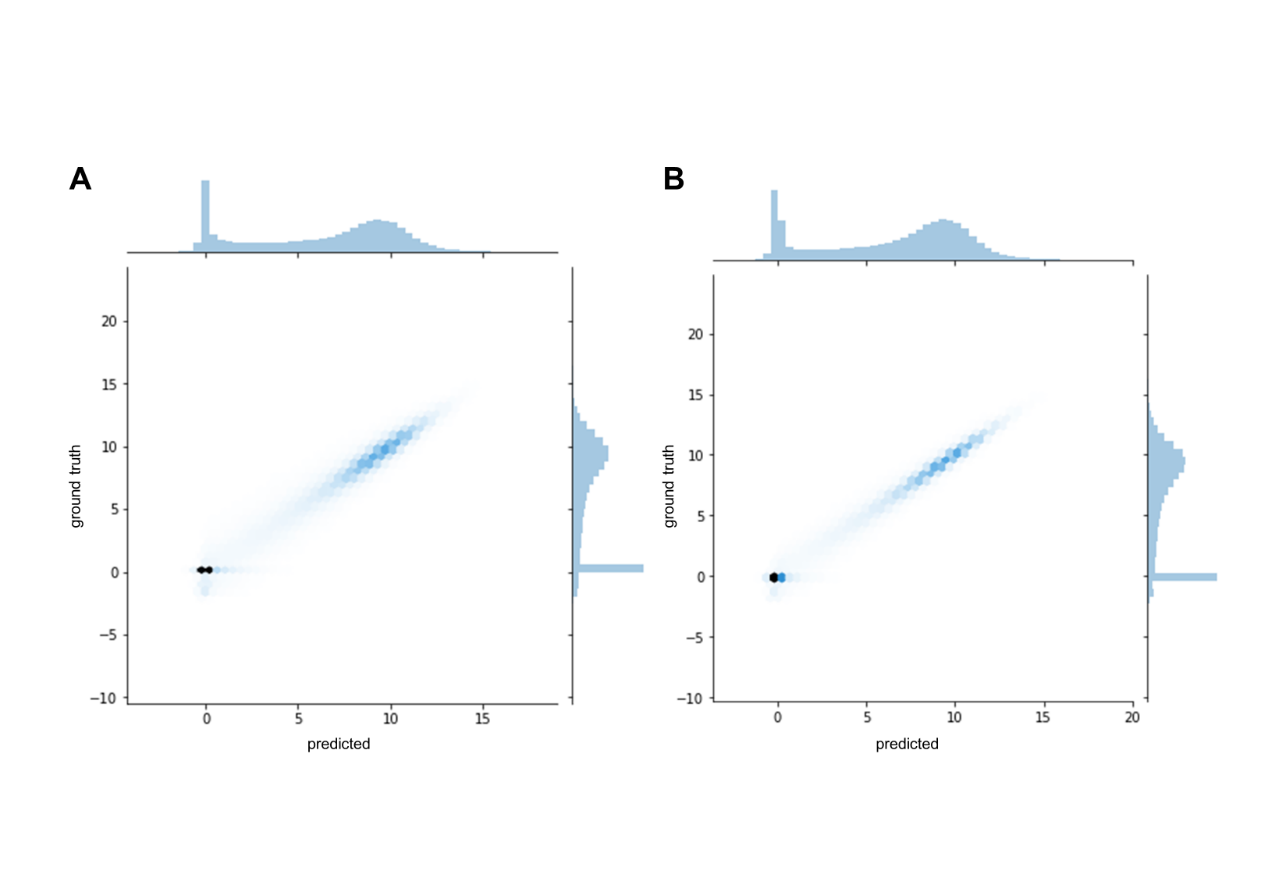

**Supplementary Figure 4.** Bivariate distribution plot of ground truth versus predicted gene expression values using (A) cancer data, and (B) normal samples data.


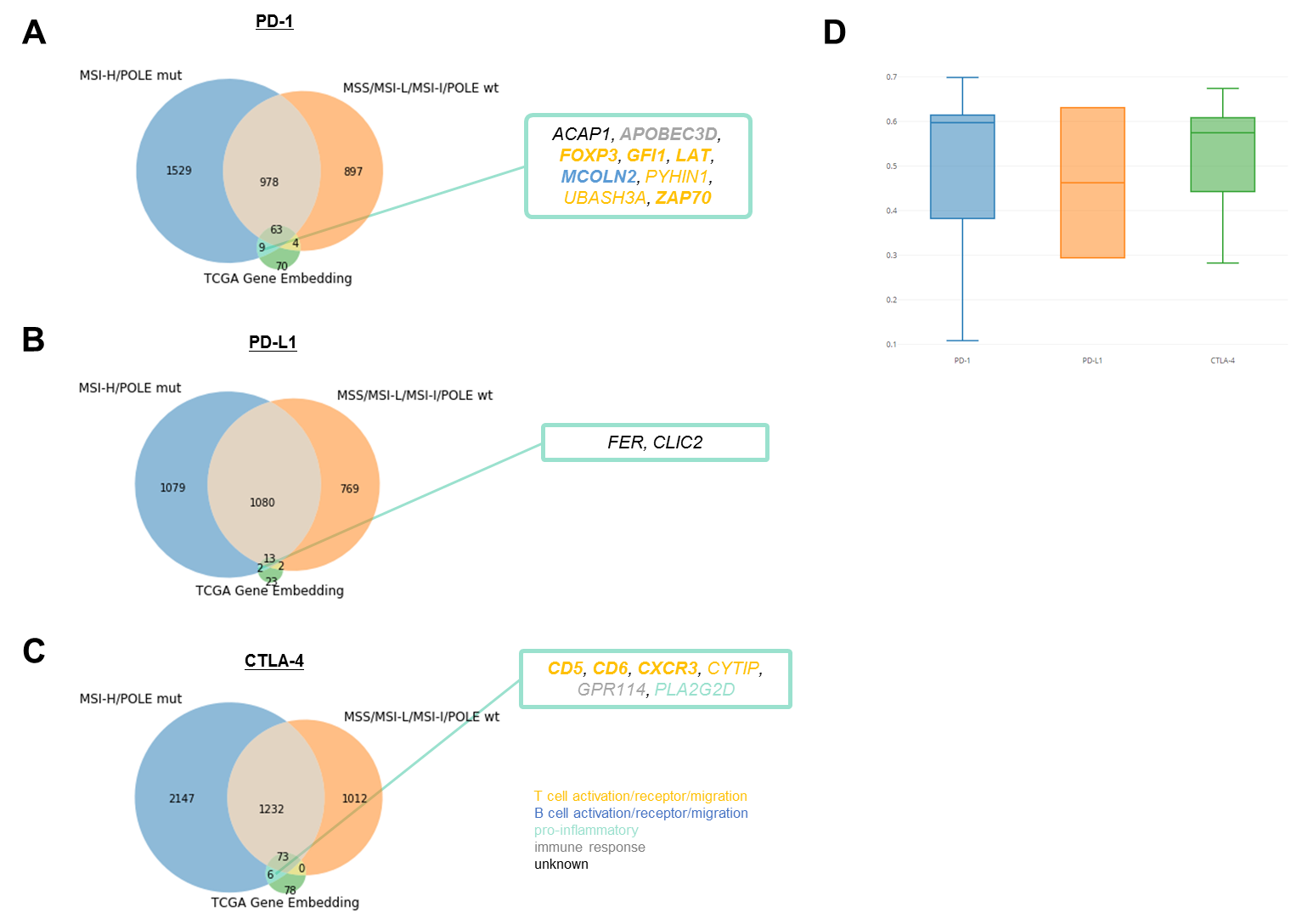
**Supplementary Figure 5**. Identification of potential related genes for immune checkpoint blockade therapy responsiveness (response stratified by microsatellite and *POLE* status).

Venn diagram of neighboring genes in simulated immunotherapy responders and non-responders with (A) PD-1, (B) PD-L1, (C) CTLA-4 and its corresponding neighbors in TCGA gene entity space. Gene relatedness supported by the literature are in bold. Genes related to T cell, B cell, pro-inflammatory or immune response are highlighted in yellow, dark blue, light blue and grey respectively. (D) Boxplot of Pearson correlation between gene candidates and respective immune checkpoint protein.

## Supplementary Table

|  | **Epoch** | **Training Loss** | **Validation Loss** |
| --- | --- | --- | --- |
| **Cancer**  **n=9544** | 0 | 2.52 | 2.42 |
|  | 1 | 1.42 | 1.55 |
|  | 2 | 1.47 | 1.43 |
| **Normal**  **n=701** | 0 | 1.96 | 2.02 |
|  | 1 | 0.88 | 0.87 |
|  | 2 | 0.84 | 0.77 |

**Supplementary Table 1**. Training and cross validation summary statistics of the three epochs in embedding model.

|  |  | Final Quantization Loss | Training Time (s) |
| --- | --- | --- | --- |
| Gene as variables | PCA initialized | 113.34 | 36.96 |
|  | random initialized | 118.47 | 28.12 |
| Sample as variables | PCA initialized | 55.89 | 9.11 |
|  | random initialized | 37.17 | 3.64 |

**Supplementary Table 2**. SOM final quantization loss and training time.

| Figure | Method | Cumulative explained variance |
| --- | --- | --- |
| 2A | 50-component PCA | 0.06% |
|  | Embedding | 61.31% |
|  | SOM, PCA initialized | 99.84% |
|  | SOM, random initialized | 99.73% |
| 2B | 50-component PCA | 0.06% |
|  | Embedding | 54.50% |
|  | SOM, PCA initialized | 53.46% |
|  | SOM, random initialized | 55.53% |

**Supplementary Table 3**. Cumulative variance explained by PC1, PC2 and PC3 in Figure 2.

|  | **r** | **Publication(DOI)** |
| --- | --- | --- |
| ***C11orf45*** | 0.16 |  |
| ***CD101*** | 0.38 | 10.4049/jimmunol.1401936 |
| ***CLEC7A*** | 0.45 | 10.1038/nm.4314 |
| ***CLIC2*** | 0.49 |  |
| ***CMPK2*** | 0.44 |  |
| ***FER*** | -0.11 |  |
| ***GNLY*** | 0.66 | 10.3389/fimmu.2017.01880 |
| ***GPR183*** | 0.52 | 10.1016/j.immuni.2015.03.002 |
| ***IL18R1*** | 0.41 | 10.7554/eLife.08133 |
| ***MCTP1*** | 0.18 |  |
| ***RASGEF1B*** | 0.40 |  |
| ***SLFN12*** | 0.24 |  |
| ***TTC39B*** | 0.12 |  |

**Supplementary Table 4**. Pearson correlation r and publications suggested the relationship of related genes with PD-1

|  | **r** | **Publication(DOI)** |
| --- | --- | --- |
| ***CD247*** | 0.55 | 10.18632/oncotarget.3642 |
| ***CD28*** | 0.39 | 10.1038/nri727 |
| ***CD3D*** | 0.53 | 10.18632/oncotarget.3642 |
| ***CD5*** | 0.49 | 10.1038/ncomms6997 |
| ***CD79B*** | 0.21 |  |
| ***CD8B*** | 0.45 | 10.18632/oncotarget.11685 |
| ***CD96*** | 0.57 | 10.1007/s00262-014-1643-7 |
| ***CFP*** | 0.23 |  |
| ***CLEC10A*** | 0.40 | 10.1128/IAI.05191-11 |
| ***CRTAM*** | 0.54 | j.jid.2017.02.292 |
| ***CST7*** | 0.42 | 10.1007/s10549-017-4281-x |
| ***CXCR2P1*** | 0.38 |  |
| ***CXCR6*** | 0.61 | 10.1097/MD.0000000000004989 |
| ***CYTIP*** | 0.59 |  |
| ***EOMES*** | 0.14 | 10.18632/oncotarget.3642 |
| ***GZMH*** | 0.55 | 10.4049/jimmunol.1401513 |
| ***GZMK*** | 0.51 | 10.1158/2326-6066.CIR-16-0087 |
| ***ITK*** | 0.53 | 10.18632/oncotarget.3642 |
| ***LAT*** | 0.23 | 10.1128/JVI.02290-10 |
| ***LTB*** | 0.41 | 10.18632/oncotarget.3216 |
| ***PTPRCAP*** | 0.42 | 10.1172/JCI91095 |
| ***TBC1D10C*** | 0.48 |  |
| ***TNFRSF8*** | 0.32 | 10.18632/oncotarget.11685 |
| ***TRAF3IP3*** | 0.50 |  |
| ***ZAP70*** | 0.41 | 10.18632/oncotarget.3642 |
| ***ZBP1*** | 0.56 | 10.1016/j.immuni.2012.08.021 |

**Supplementary Table 5.** Pearson correlation r and publications suggested the relationship of related genes with PD-L1.

|  | **r** | **Publication(DOI)** |
| --- | --- | --- |
| ***ACAP1*** | 0.64 |  |
| ***CCR5*** | 0.64 | 10.1038/bjc.2014.572 |
| ***CD2*** | 0.66 | 10.1007/s00262-008-0627-x |
| ***CD247*** | 0.64 | 10.1007/s00262-016-1849-y |
| ***CD27*** | 0.64 | 10.1007/s00262-008-0507-4 |
| ***CD28*** | 0.52 | 10.1016/S0092-8674(05)80059-5 |
| ***CD3D*** | 0.65 | 10.1371/journal.pgen.1006477 |
| ***CD5*** | 0.63 | 10.1007/s00262-008-0627-x |
| ***CD6*** | 0.60 | 10.1007/s00262-015-1712-6 |
| ***CD8B*** | 0.57 | 10.1158/0008-5472.CAN-06-2379 |
| ***CD96*** | 0.63 | 10.1016/j.coi.2012.01.009 |
| ***CST7*** | 0.64 | 10.1007/s10549-017-4281-x |
| ***CTSW*** | 0.60 | 10.1186/s40425-017-0215-8 |
| ***CXCL11*** | 0.52 | 10.1155/2011/865684 |
| ***CXCR3*** | 0.64 | 10.1155/2011/865684 |
| ***CXCR6*** | 0.67 | 10.1016/j.eururo.2011.10.035 |
| ***CYTIP*** | 0.59 |  |
| ***FCRL5*** | 0.46 | 10.1016/j.celrep.2015.09.070 |
| ***FCRLA*** | 0.25 | 10.1016/j.ab.2013.07.032 |
| ***GZMA*** | 0.61 | 10.1007/s10549-017-4281-x |
| ***GZMH*** | 0.63 | 10.1186/s40425-017-0215-8 |
| ***ITK*** | 0.72 | 10.1038/nm.3393 |
| ***KLRK1*** | 0.65 | 10.4161/onci.23127 |
| ***LCK*** | 0.63 | 10.1371/journal.pgen.1006477 |
| ***LTB*** | 0.55 | 10.1038/ni1029 |
| ***MAP4K1*** | 0.52 | 10.1177/1010428317707882 |
| ***MGC29506*** | 0.47 | 10.1016/j.humimm.2018.02.009 |
| ***MMP25*** | 0.50 |  |
| ***PTPRCAP*** | 0.62 | 10.1200/JCO.2018.36.15_suppl.12104 |
| ***SLAMF6*** | 0.65 | 10.1158/0008-5472.CAN-10-2229 |
| ***TNFRSF8*** | 0.54 | 10.1016/j.cell.2017.08.004 |
| ***TNFRSF9*** | 0.61 | 10.1172/JCI46102 |
| ***TRAF3IP3*** | 0.64 |  |
| ***ZAP70*** | 0.60 | 10.4049/jimmunol.164.1.49 |

**Supplementary Table 6.** Pearson correlation r and publications suggested the relationship of related genes with CTLA-4.
